# Gait analysis may distinguish progressive supranuclear palsy and Parkinson disease since the earliest stages

**DOI:** 10.1038/s41598-021-88877-2

**Published:** 2021-04-29

**Authors:** Marianna Amboni, Carlo Ricciardi, Marina Picillo, Chiara De Santis, Gianluca Ricciardelli, Filomena Abate, Maria Francesca Tepedino, Giovanni D’Addio, Giuseppe Cesarelli, Giampiero Volpe, Maria Consiglia Calabrese, Mario Cesarelli, Paolo Barone

**Affiliations:** 1 Center for Neurodegenerative Diseases (CEMAND), Department of Medicine, Surgery and Dentistry “Scuola Medica Salernitana”, University of Salerno, Via Salvador Allende, 43, 84081 Baronissi, SA Italy; 2 IDC Hermitage-Capodimonte, Naples, Italy; 3 Department of Advanced Biomedical Sciences, University of Naples “Federico II”, Naples, Italy; 4 Istituti Clinici Scientifici Maugeri IRCCS, Pavia, Italy; 5 Azienda Ospedaliera Universitaria OO. RR. San Giovanni di Dio e Ruggi d’Aragona, Salerno, Italy; 6 Department of Chemical, Materials and Production Engineering, University of Naples “Federico II”, Naples, Italy; 7 Department of Electrical Engineering and Information Technology, University of Naples “Federico II”, Naples, Italy

**Keywords:** Neuroscience, Biomarkers, Medical research, Neurology

## Abstract

Progressive supranuclear palsy (PSP) is a rare and rapidly progressing atypical parkinsonism. Albeit existing clinical criteria for PSP have good specificity and sensitivity, there is a need for biomarkers able to capture early objective disease-specific abnormalities. This study aimed to identify gait patterns specifically associated with early PSP. The study population comprised 104 consecutively enrolled participants (83 PD and 21 PSP patients). Gait was investigated using a gait analysis system during normal gait and a cognitive dual task. Univariate statistical analysis and binary logistic regression were used to compare all PD patients and all PSP patients, as well as newly diagnosed PD and early PSP patients. Gait pattern was poorer in PSP patients than in PD patients, even from early stages. PSP patients exhibited reduced velocity and increased measures of dynamic instability when compared to PD patients. Application of predictive models to gait data revealed that PD gait pattern was typified by increased cadence and longer cycle length, whereas a longer stance phase characterized PSP patients in both mid and early disease stages. The present study demonstrates that quantitative gait evaluation clearly distinguishes PSP patients from PD patients since the earliest stages of disease. First, this might candidate gait analysis as a reliable biomarker in both clinical and research setting. Furthermore, our results may offer speculative clues for conceiving early disease-specific rehabilitation strategies.

## Introduction

Progressive supranuclear palsy (PSP) is a rare and rapidly progressing neurodegenerative disease classified among atypical Parkinsonisms, with a prevalence of 5–6 cases per 100,000^1^. PSP Richardson’s syndrome, the most frequent form of the disease, is characterized by vertical supranuclear gaze palsy and postural instability with early falls^2^. Extant evidence suggests that the clinical spectrum of PSP is larger than originally described. In particular, the second most common form of disease, accounting for a third of cases, is characterized by a parkinsonian syndrome resembling Parkinson’s disease (PD) especially in the earliest stages^3^.

Recently, several PSP variants were detailed in the International Parkinson and Movement Disorder Society criteria for diagnosis of PSP (MDS-PSP)^4^ and subsequently characterized in real-life clinical settings^5,6^.

Although semi-quantitative rating scales such as the Movement Disorder Society Unified Parkinson’s Disease Rating Scale (MDS-UPDRS)^7^ and PSP Rating Scale (PSP-RS)^8^ provide a clinician-based quantification of disease burden, these scales do not provide objective quantitative measures. Indeed, there is a need to employ quantitative tools for evaluating motor function in parkinsonism^9^; among those, gait analysis is one of the main instruments used to assess locomotion. Gait analysis is a non-invasive, 3-dimensional computerized examination of gait, commonly used in the literature to investigate and distinguish various diseases^10^.

Gait analysis has been employed for different objectives in PD patients, including the investigation of pathophysiological mechanisms underpinning the disease, evaluation of treatment outcomes, automatic recognition of PD symptoms, and implementation of algorithms for PD diagnosis and staging^11–14^. In addition, gait analysis has been used to explore the association between specific gait patterns and specific symptoms of PD, such as mild cognitive impairment^15,16^ and freezing of gait^17^.

Quantitative tools for assessing locomotion have also been applied in PSP patients. Amano et al. ^18^ examined the biomechanical features of dynamic postural control during gait initiation and ambulation in PSP patients using a gait analysis system. Hatanaka et al. compared the gait features of PSP, PD patients, and controls using a portable triaxial accelerometer rhythmogram^19^. Other studies have employed sensor-based approaches. In particular, Raccagni et al. investigated the ability of a gait assessment system to detect differences in gait parameters in atypical parkinsonian disorders^20^, whereas Gaβner et al. assessed whether sensor-based gait parameters could serve as a complementary tool to clinical scores for distinguishing atypical parkinsonism from PD^21^. More recently, machine-learning approaches have been introduced to assess the gait patterns of PSP patients, although the rarity of this disease poses challenges to obtaining large datasets for analysis. In our previous work, we distinguished de novo PD, stable PD, and PSP patients using machine-learning techniques applied to gait parameters after artificial data augmentation and achieved promising results^22^. Subsequently, De Vos et al. performed a similar study using wearable technology^23^.

Albeit the MDS-PSP criteria^4^ have recently proven to have good specificity and sensitivity^24^, there is a need for biomarkers able to capture early objective disease-specific abnormalities and to monitor disease progression over time. The main aim of the present study was to identify gait patterns specifically associated with PSP with two-fold impact: (1) providing a proof of concept that quantitative gait evaluation may represent a reliable biomarker since the earliest stages of disease; (2) recognizing early disease-specific gait patterns useful to design tailored rehabilitation programs.

## Methods

### Study design and population

The study population consisted of 104 participants (83 PD and 21 PSP patients) consecutively enrolled between February 2018 and July 2020. Participants were selected from patients referred to the Movement Disorders Unit of the Institute for Diagnosis and Care Hermitage-Capodimonte of Naples and Center for Neurodegenerative Diseases of the University of Salerno. All PD patients fulfilled the Movement Disorder Society (MDS) clinical diagnostic criteria for PD^25^. Newly diagnosed PD patients presented with symptom onset within 1 year from enrolment and were included after undergoing a [^123^I]FP-CIT SPECT examination for dopamine transporter assessment which indicated nigro-striatal degeneration. All PSP patients met the clinical diagnostic criteria proposed by MDS^4,26^ and qualified for diagnosis of probability. Of patients, 11 (52.3%) presented with Richardson’s syndrome. The remaining patients presented with other variant syndromes of PSP (five patients exhibited PSP with predominant parkinsonism and four patients exhibited PSP with predominant gait freezing). Of the PD patients, 56 were stable; i.e., they received stable treatment during the 4 weeks preceding enrolmentt, and 27 were newly diagnosed PD patients who had never received treatment. Of the PSP patients, 12 were early PSP patients (disease duration less than 2 years). The exclusion criteria for all patients were as follows: gait requiring assistance; dementia according to the DSM-V criteria; clinically significant comorbidities, including other neurologic disorders, orthopedic diseases, or cardiovascular/respiratory diseases; anticholinergic or neuroleptic treatment; and/or brain surgery. All participants were evaluated using an assessment including demographic, clinical, and anthropometric data. All participants were evaluated in the self-defined best “on-state” while receiving their typical dopaminergic drugs.

### Standard protocol approvals, registrations, and patient consent

This study was performed in accordance with the 1964 Declaration of Helsinki and was approved by Campania Sud, the reference ethics committee of the Center for Neurodegenerative Diseases of the University of Salerno. Written informed consent was obtained from all participants.

### Gait analysis

Gait analysis was performed in all subjects using a BTS Bioengineering system. The SMART DX is an optical system equipped with six infrared cameras, two video cameras, two force plates, a set of passive markers, and an elaborator. The Davis protocol was used for all subjects^27^, comprising the following phases. Anthropometric measurements of the patients (height, weight, leg length, etc.) were obtained. In total, 22 reflective markers were positioned on specific points of the body. The standing phase consisted of assessments of the patient while standing up on a force plate. This was followed by the walking phase on a 10-m path. All patients were evaluated on the straight pathway during two different tasks: (1) GAIT: normal gait, namely the single task; (2) COG: walking while serially subtracting 7 s starting from 100, namely the dual task; each task was performed four times. Prior to commencing the trials, all participants were trained to walk at a normal pace at their usual speed, without any instructions to prioritize walking or calculating. This procedure generated a report from which spatial and temporal parameters were extracted.

### Statistical analysis

IBM SPSS v.25 was used to perform all the statistical analyses. For univariate statistical analysis, the Shapiro Wilk and Kolmogorov Smirnov tests were used to assess normality according to the sample size (the former for n < 50, and the latter for n > 50). For normally distributed data, the Levene test was used to assess the homoscedasticity of the variances between the compared groups. A t-test for independent samples was employed when both of the previous assumptions were verified; a Mann Whitney test was otherwise employed. Univariate statistical analysis was performed to quantify the effects of the COG task on the two groups. Binary logistic regression^28^ was computed to produce models capable of classifying patients into a diagnostic group (PD or PSP) starting from spatial and temporal parameters of gait. The presence of multicollinearity among variables and outliers was verified. The former was assessed by computing the coefficients of correlation, and all variables with a correlation greater than 0.80 were removed; the latter was verified by computing the a-dimensional Cook’s distance and Center Leverage Value. The odds ratios with a confidence interval of 95% and relative p-values were provided for each variable included in the models. The Hosmer Lemeshow goodness-of-fit test was computed to evaluate whether the observed event rates matched expected event rates in subgroups of the model population. Finally, the overall accuracy of the models and capacity to detect each group were determined. Alpha significance level was set to p < 0.05 for all statistical analyses.

## Results

Univariate statistical analysis and binary logistic regression were performed twice: first, all PD patients (N = 83) were compared with all PSP patients (N = 21). Subsequently, the analysis was restricted to newly diagnosed PD patients (N = 27) and early PSP patients (N = 12).

### PD versus PSP

#### Univariate statistical analysis

Univariate statistical analysis comparing demographic and clinical features, and spatial and temporal gait parameters for both GAIT and COG tasks between PD and PSP patients are presented in Tables 1 and 2, respectively.

**Table 1:** Comparison of demographic and clinical features between PD and PSP patients.

Feature	PD	PSP	p-value	Newly diagnosed PD	Early PSP	p-value
Age	63.27 ± 8.54	67.81 ± 7.48	0.027*	63.33 ± 8.74	63.50 ± 5.96	0.845
Gender (M/F)	55/28	11/10	0.238	17/10	7/5	0.784
Disease duration (years)	3.46 ± 3.20	2.52 ± 1.17	0.496	< 1	1.75 ± 0.43	NA
Levodopa Equivalent Daily dose (mg)	377.18 ± 39 6.70	409.44 ± 258.9	0.411	0.00 ± 0.00	319.00 ± 177 80	NA
Body Mass Index	27.17 ± 3.38	28.33 ± 4.68	0.265	26.18 ± 3.06	26.73 ± 4.61	0.849
Hoehn & Yahr	1.69 ± 0.47	2.32 ± 0.50	< 0.001*	1.42 ± 0.50	2.22 ± 0.47	0.001*
MDS-UPDRS: part III	19.46 ± 8.99	-	NA	12.85 ± 6.17	–	NA
PSP-RS V	–	5.81 ± 2.73	NA	–	5,.3 ± 2.87	NA
PSP-RS VI	–	7.28 ± 4.55	NA	–	6.58 ± 3.73	NA
Mini-mental state examination	26.87 ± 2.31	25.16 ± 3.06	0.003*	26.92 ± 2.21	25.58 ± 2.75	0.071

*NA* not applicable, *PD* Parkinson’s disease, *PSP* progressive supranuclear palsy, *MDS-UPDRS* movement disorder society unified Parkinson’s disease rating scale, *PSP-RS* progressive supranuclear palsy rating scale.

*significance level at 0.05.

**Table 2:** Univariate statistical analysis comparing all gait parameters (mean ± SD) between PD and PSP patients.

Features	Measure	GAIT task			COG dual task		
		PD	PSP Mean ± SD	p-value	PD Mean ± SD	PSP Mean ± SD	p-value
Cycle duration	s	1.10 ± 0.11	1.29 ± 0.23	< 0.0001***	1.16 ± 0.14	1.42 ± 0.28	< 0.0001***
Stance duration	s	0.67 ± 0.08	0.82 ± 0.16	< 0.0001***	0.72 ± 0.09	0.94 ± 0.22	< 0.0001***
Swing duration	s	0.37 ± 0.10	0.27 ± 0.08	< 0.0001***	0.26 ± 0.08	0.26 ± 0.05	0.221
Variability of swing duration	s	0.07 ± 0.06	0.17 ± 0.13	< 0.0001***	0.13 ± 0.04	0.16 ± 0.04	< 0.0001***
Stance phase	%	60.27 ± 1.97	62.84 ± 2.79	< 0.0001***	61.95 ± 2.20	65.64 ± 3.51	< 0.0001***
Swing phase	%	39.54 ± 1.70	37.01 ± 2.89	< 0.0001***	38.54 ± 3.62	34.36 ± 3.51	< 0.0001***
Single support phase	%	39.45 ± 2.12	37.05 ± 2.95	< 0.0001***	38.08 ± 2.54	34.36 ± 3.51	< 0.0001***
Double support phase	%	10.82 ± 2.95	15.05 ± 6.58	< 0.0001***	12.64 ± 3.10	17.47 ± 5.02	< 0.0001***
Mean velocity	m/s	1.04 ± 0.16	0.68 ± 0.26	< 0.0001***	0.89 ± 0.19	0.52 ± 0.23	< 0.0001***
Mean velocity	%height/s	62.07 ± 10.31	41.69 ± 15.00	< 0.0001***	53.58 ± 10.95	31.79 ± 13.53	< 0.0001***
Cadence^	Steps/min	109.61 ± 11.52	95.80 ± 17.27	0.002**	104.63 ± 12.30	87.99 ± 17.51	0.001**
Cycle length	m	1.13 ± 0.14	0.84 ± 0.21	< 0.0001***	1.02 ± 0.17	0.69 ± 0.22	< 0.0001***
Cycle length	%height	67.77 ± 8.82	51.70 ± 12.54	< 0.0001***	61.47 ± 10.79	42.59 ± 12.95	< 0.0001***
Step length	m	0.46 ± 0.15	0.25 ± 0.13	< 0.0001***	0.33 ± 0.10	0.24 ± 0.17	< 0.0001***
Variability of step length	m	0.27 ± 0.42	0.31 ± 0.51	0.105	0.24 ± 0.20	0.21 ± 0.30	< 0.0001***
Step width	m	0.10 ± 0.07	0.11 ± 0.04	0.156	0.11 ± 0.10	0.12 ± 0.06	0.088

*PD* Parkinson’s disease, *PSP* progressive supranuclear palsy.

**Significance level at 0.01.

***Significance level at 0.001.

^Normally distributed according to the Shapiro Wilk test (homoscedasticity verified for all t-tests) and t-test applied; the other data were analyzed with a Mann Whitney test.

In the GAIT task, PSP patients exhibited poorer gait patterns when compared to PD patients. Namely, relative to PD patients, PSP patients exhibited reduced velocity and cadence, shortened step and cycle lengths, increased cycle duration mainly due to longer double support stance phase duration, and increased swing duration variability (Table 2).

In the COG task, PSP patients exhibited the same gait features as those displayed during the single task, with the exception of two gait variables, namely swing duration and step length variability (Table 2). For both GAIT and COG tasks, the difference in step width between the two groups was not statistically significant. When comparing the effect of the dual task on gait measures in PD and PSP patients, the simultaneous performance of the secondary task significantly worsened most gait measures in both groups with the exception of the step width that was significantly increased in PSP patients but not in PD patients (Table S1).

#### Binary logistic regression

Binary logistic regression was performed to distinguish PD and PSP patients. The results for GAIT and COG tasks are presented in Table 3.

**Table 3:** Multinomial logistic regression results for GAIT and COG tasks to distinguish PD and PSP.

Variables	GAIT task		COG dual task	
	Odds ratio (95% CI)	p-value	Odds ratio (95% CI)	p-value
Cycle duration	NI	NI	NI	NI
Stance duration	NI	NI	NI	NI
Swing duration	0.000 (0.000–0.009)	0.010**	NI	NI
Variability of swing’s duration	NI	NI	NI	NI
Stance phase	1.429 (1.192–1.713)	< 0.0001***	1.396 (1.129–1.726)	0.002**
Swing phase	NI	NI	NI	NI
Single support phase	NI	NI	NI	NI
Double support phase	NI	NI	NI	NI
Mean velocity	NI	NI	NI	NI
Mean velocity	NI	NI	NI	NI
Cadence^	0.922 (0.870–0.977)	0.006**	0.891 (0.811–0.978)	0.015*
Cycle length	0.000 (0.000–0.26)	0.002**	0.000 (0.000–0.004)	0.001**
Cycle length	NI	NI	NI	NI
Step length	NI	NI	NI	NI
Variability of step length	NI	NI	NI	NI
Step width	NI	NI	NI	NI

Odds ratio (95% confidence interval) and p-value for each variable were considered in the model.

*PD* Parkinson’s disease, *PSP* progressive supranuclear palsy, *NI* not included.

*Significance level at 0.05.

**significance level at 0.01.

***significance level at 0.001.

Two outliers were removed from the GAIT model, and five outliers were removed from the COG model. The graphs depicting Cook’s distance versus center leverage values are presented in the supplemental data (Figs. S1 and S2). The overall accuracies for the GAIT and COG models were 92.3% and 95%, respectively. The capacities to detect PD patients were 96.5% and 96.4%, and the capacities to identify PSP patients were 73.7% and 88.2% for the GAIT and COG models, respectively. The positive predictive value and the negative predictive value were 82.3% and 94.2% for the GAIT task, 83.3% and 97.6% for the COG task. Table 4 shows the confusion matrix of each model.

**Table 4:** Confusion matrix of binary logistic regression (PD Vs PSP) for GAIT and COG tasks.

GAIT		Predicted	
		PD	PSP
Observed	PD	82	3
	PSP	5	14

COG		Predicted	
		PD	PSP
Observed	PD	80	3
	PSP	2	15

*PD* Parkinson’s disease, *PSP* progressive supranuclear palsy.

The odds ratios of the variables included in the GAIT model revealed that longer swing duration and increased cadence and cycle length were associated with a higher probability of being in the PD group. Conversely, longer stance phase was associated with a higher probability of being in the PSP group. The COG model confirmed these results, with the exception of swing duration, which was not entered in this model. The Hosmer Lemeshow goodness-of-fit test demonstrated good overall quality of the models, with p-values of 0.976 and 1.000 for the GAIT model and COG model, respectively.

### Newly diagnosed PD versus early PSP

#### Univariate statistical analysis

Univariate statistical analysis was performed to compare spatial and temporal parameters of gait between newly diagnosed PD and early PSP patients. The results for GAIT and COG tasks are presented in Table 5.

**Table 5:** Univariate statistical analysis comparing all gait parameters (mean ± SD) between newly diagnosed PD and early PSP patients.

Features	Measure	GAIT task			COG dual task		
		Newly diagnosed PD	Early PSP	p-value	Newly diagnosed PD	Early PSP	p-value
Cycle duration	s	1.12 ± 0.13	1.34 ± 0.24	0.002**	1.17 ± 0.11	1.45 ± 0.24	0.001**
Stance duration	s	0.68 ± 0.10	0.85 ± 0.17	0.002**	0.73 ± 0.09	0.95 ± 0.19	0.001**
Swing duration	s	0.44 ± 0.04	0.49 ± 0.07	0.017*	0.44 ± 0.03	0.49 ± 0.07	0.056
Variability of swing duration	s	0.03 ± 0.02	0.12 ± 0.26	0.061	0.04 ± 0.02	0.06 ± 0.02	0.007**
Stance phase^	%	60.48 ± 1.82	63.13 ± 2.38	< 0.0001***	61.93 ± 1.76	65.62 ± 3.29	< 0.0001***
Swing phase^	%	39.52 ± 1.82	36.78 ± 2.30	< 0.0001***	38.07 ± 1.76	34.38 ± 3.29	< 0.0001***
Single support phase	%	39.49 ± 1.80	36.88 ± 2.38	0.001**	38.09 ± 1.76	34.35 ± 3.32	< 0.0001***
Double support phase	%	11.33 ± 3.38	13.05 ± 2.24	0.014*	12.04 ± 1.75	16.11 ± 3.82	0.001**
Mean velocity^	m/s	1.04 ± 0.18	0.73 ± 0.29	< 0.0001***	0.91 ± 0.16	0.58 ± 0.26	< 0.0001***
Mean velocity^	%height/s	62.38 ± 11.66	44.31 ± 16.89	< 0.0001***	55.24 ± 9.29	35.34 ± 15.52	< 0.0001***
Cadence^	steps/min	108.65 ± 11.45	92.47 ± 16.93	0.001**	103.52 ± 9.90	85.68 ± 15.38	0.001**
Cycle length^	m	1.14 ± 0.14	0.92 ± 0.20	< 0.0001***	1.06 ± 0.12	0.79 ± 0.22	< 0.0001***
Cycle length^	%height	68.54 ± 8.04	56.17 ± 11.66	< 0.0001***	63.75 ± 7.09	47.99 ± 13.38	0.001**
Step length	m	0.53 ± 0.12	0.43 ± 0.13	0.018*	0.50 ± 0.10	0.37 ± 0.14	0.004**
Step length variability	m	0.21 ± 0.42	0.25 ± 0.71	0.298	0.20 ± 0.34	0.30 ± 0.77	0.086
Step width	m	0.11 ± 0.10	0.08 ± 0.02	0.480	0.10 ± 0.08	0.10 ± 0.03	0.233

*PD* Parkinson’s disease, *PSP* progressive supranuclear palsy, *SD* standard deviation.

*Significance level at 0.05.

**Significance level at 0.01.

***Significance level at 0.001.

^Normally distributed according to the Shapiro Wilk test (homoscedasticity verified for all t-tests) and t-test applied; the other data were analyzed with a Mann Whitney test.

For the single task (GAIT), early phase PSP patients exhibited poorer walking parameters when compared with PD patients. In particular, compared to newly diagnosed PD patients, early PSP patients exhibited reduced velocity and cadence, shortened step and cycle length, and increased cycle duration; these patients tended to rely on a longer double support stance phase (Table 5).

In the dual task (COG), compared to newly diagnosed PD patients, early PSP patients exhibited a gait pattern similar to that during the single task with the exception of two gait variables, namely, swing duration and swing duration variability (Table 5). For both GAIT and COG tasks, the differences in step length variability and step width between the two groups were not statistically significant. When comparing the effects of the dual task on gait measures in newly diagnosed PD patients versus early PSP patients, most gait parameters were similarly altered in the dual task condition in both groups, with the exception of step length variability and step width, which were influenced by the dual task in PSP patients but not in PD patients (Table S2).

#### Binary logistic regression

The results of the binary logistic regression conducted to differentiate newly diagnosed PD patients and early PSP patients are presented in Table 6 for both GAIT and COG tasks.

**Table 6:** Multinomial logistic regression results for GAIT and COG tasks to distinguish newly diagnosed PD and early PSP patients.

Variables	GAIT task		COG dual task	
	Odds Ratio (95% CI)	p-value	Odds Ratio (95% CI)	p-value
Cycle duration	NI	NI	NI	NI
Stance duration	NI	NI	NI	NI
Swing duration	NI	NI	NI	NI
Variability of swing’s duration	NI	NI	NI	NI
Stance phase	1.303 (1.087–1.561)	0.040*	1.280 (1.072–1.529)	0.006**
Swing phase	NI	NI	NI	NI
Single support phase	NI	NI	NI	NI
Double support phase	NI	NI	NI	NI
Mean velocity	NI	NI	NI	NI
Mean velocity	NI	NI	NI	NI
Cadence^	0.960 (0.893–1.033)	0.274	0.843 (0.735–0.967)	0.014*
Cycle length	0.000 (0.000–0.143)	0.021*	NI	NI
Cycle length	NI	NI	NI	NI
Step length	NI	NI	0.078 (0.000–1630.00)	0.615
Variability of the length of step	NI	NI	NI	NI
Step width	NI	NI	NI	NI

Odds ratio (95% confidence interval) and p-value for each variable were considered in the model.

*PD* Parkinson’s disease, *PSP* progressive supranuclear palsy, *NI* not included.

*Significance level at 0.05.

**Significance level at 0.01.

Two outliers were removed from each of the two models. The graphs depicting Cook’s distance versus center leverage values are presented in the supplemental data (Figs. S3 and S4). For the GAIT and COG models. The overall accuracies were 89.2% and 91.9% for the GAIT and COG models, respectively. The capacities to detect newly diagnosed PD patients were 92.3% and 96.3%, and the capacities to identify early PSP patients were 81.8% and 80.0% for the GAIT and COG models, respectively. The positive predictive value and the negative predictive value were 81.8% and 92.3% for the GAIT task, 88.9% and 96.2% for the COG task. Table 7 shows the confusion matrix of each model.

**Table 7:** Confusion matrix of binary logistic regression (newly diagnosed PD vs early PSP) for GAIT and COG tasks.

GAIT		Predicted	
		PD	PSP
Observed	PD	24	2
	PSP	2	9

COG		Predicted	
		PD	PSP
Observed	PD	26	1
	PSP	1	8

*PD* Parkinson’s disease, *PSP* progressive supranuclear palsy.

The odds ratios of the variables included in the GAIT model revealed that longer cycle length was associated with a higher probability of being in the newly diagnosed PD group. In contrast, longer stance phase was associated with a higher probability of being in the early PSP group. The COG model confirmed the same predictor, i.e. longer stance phase, for PSP diagnosis, whereas it disclosed that increased cadence raises the probability of PD diagnosis. The Hosmer Lemeshow goodness-of-fit test demonstrated good overall quality of the models, with p-values of 0.846 and 0.195 for the GAIT model and COG model, respectively.

## Discussion

Here, we demonstrated that PSP patients exhibited disease-specific gait pattern when compared to PD patients, even during the earliest stages of disease. In particular, PSP patients exhibited reduced velocity and increased measures of dynamic instability when compared to PD subjects. Furthermore, application of predictive models to gait data revealed that increased cadence and longer cycle length characterized PD gait pattern, whereas longer stance phase distinguished PSP subjects in both mid and early stages.

### Comparison of gait patterns in PD and PSP patients

Itn the single task, PSP subjects exhibited reduced velocity and cadence, shortened step and cycle length, increased cycle duration (mainly due to longer double support stance phase), and increased swing time variability when compared to PD patients. These data indicating that PSP gait pattern is characterized by dynamic instability are consistent with previous findings in smaller samples^19,21,29^. These results suggest that complex dysfunction of internal motor programming is more prominent in PSP patients than in PD patients, with the former group exhibiting dysfunction in both spatial and temporal domains, and the latter group exhibiting predominantly spatial dysfunction^30,31^. Notably, because dysfunction of spatial gait variables are generally responsive to dopaminergic treatment in PD patients^32^, the different gait patterns of the two patient groups could at least partly depend on the effectiveness of medication that is notoriously poor in PSP patients^4^. Gait patterns of the two groups were similar in both the single and dual tasks, with non-specific detrimental effects of the secondary task on both PSP and PD patients. Importantly, the major effect of the dual task in PSP patients was an increase in step width that could represent an attempt to counteract the lateral instability commonly observed as balance and walking deficits in PSP patients^19^.

Application of predictive models on the single task data revealed that longer swing duration and increased cadence and cycle length were characteristic gait features of PD patients. In contrast, longer stance phase was a gait feature that typified the gait pattern of PSP patients. Data from the dual task condition confirmed the single task data with the exception of swing duration. These results indicate that relative to PSP patients, PD patients display more stable locomotion, even under the dual task condition, and exhibit superior performance in temporal gait parameters that thus represent the variables better discriminating between PD and PSP^30,31^.

### Comparison of gait patterns in newly diagnosed PD versus early PSP patients

Whole-group differences were recapitulated in the comparison of gait features between newly diagnosed PD and early PSP patients. From early stages, patients with PSP exhibited reduced velocity and cadence, shortened step and cycle length, and increased cycle duration which was predominantly underpinned by a longer double support stance phase. These findings were independent of dopaminergic effects, since newly diagnosed PD patients were drug-naïve. The dual task condition significantly modified most gait measures in both patients groups. Notably, step length variability and step width were influenced by the dual task in PSP patients but not in PD patients. These data highlight two important points. First, early stage PD and PSP patients exhibit distinct gait patterns, which likely reflect different underlying pathophysiological mechanisms. Second, in the early stage, the dual task condition may be more detrimental in PSP patients than in PD patients. Our findings are consistent with the recent observation that gait impairments in PSP are associated with an imbalance in the control of indirect and direct locomotor pathways. In particular, PSP patients display specific dysfunction of the indirect prefrontal–subthalamic–pedunculopontine loop of locomotor control, which drives modulated gait, and increased activity in the direct loop, which regulates stereotyped gait^33^. Given that the dual task condition mainly involves cognitive-mediated gait^34^ that is underpinned by the indirect pathway, it is unsurprising that PSP patients exhibit greater effects in the dual task condition, at least in the early stage. With disease progression, this effect tends to be masked by the general deterioration of all gait features, hence the loss of disease specificity of the dual task effect in more advanced patients, as reported above.

Application of predictive models on single task data revealed that increased cycle length was a gait variable characteristic of newly diagnosed PD patients, whereas longer stance phase was a gait feature that typified early PSP gait pattern. In the dual task condition, longer stance phase was confirmed as the best predictor for PSP diagnosis, whereas increased cadence was the strongest predictor for PD diagnosis. These findings indicate that from an early stage, PSP patients exhibit more alterations in temporal gait features when compared to PD patients. Further, they suggest that the dual task condition exerts a major effect on step length^35^ in PD patients from a very early stage, as reflected by increased cadence.

The present study has some limitations. First, the sample size of PSP patients was relatively small, mainly due to the low prevalence of the disease and the early loss of independent walking. Second, when comparing gait patterns in newly diagnosed PD and early PSP patients, we indeed confronted patients with (PSP) and without (PD) dopaminergic treatment. Nevertheless, given the poor response to dopaminergic treatment in PSP patients^4^ and the general confirmation of the different gait patterns between the two patients groups in both early and mid-stages of disease, we hypothesize that medication status had a minimal impact on our findings. Finally, we admit that the PSP group included different phenotypes and the “stable” PD group consisted of patients in slightly different Hoehn and Yahr stages; nevertheless, predictive models were able to capture disease-specific gait patterns.

In conclusion, the present study demonstrates that quantitative gait evaluation clearly distinguishes PSP patients from PD patients since the earliest stages of disease. Our findings indicate that gait analysis could be candidate as a reliable biomarker in both clinical and research setting. In addition, our results may offer speculative clues for conceiving early disease-specific rehabilitation strategies.

## Supplementary Information

Supplementary Information.
