# Supplementary material for: Gait analysis may distinguish progressive supranuclear palsy and Parkinson disease since the earliest stages
Source: Sci Rep. 2021 Apr 29;11:9297. doi: 10.1038/s41598-021-88877-2 (PMC8084977; doi:10.1038/s41598-021-88877-2)
Supplement: Supplementary file 1 — Supplementary Information. [file 41598_2021_88877_MOESM1_ESM.pdf]

Gait analysis may distinguish Progressive Supranuclear Palsy and Parkinson Disease since the earliest stages

Marianna Amboni[1,2]* MD, PhD, Carlo Ricciardi[3,4] MSc, Marina Picillo[1] MD, PhD, Chiara De Santis[1] MD, Gianluca Ricciardelli[5] PT, Filomena Abate[1] MD, Maria Francesca Tepedino[1] MD, Giovanni D'Addio[4] MSc, Giuseppe Cesarelli[4,6] PhD, Giampiero Volpe[5] MD, Maria Consiglia Calabrese[1] PT, PhD, Mario Cesarelli[4,7] MSc, Paolo Barone[1] MD, PhD.

[1]Center for Neurodegenerative Diseases (CEMAND), Department of Medicine, Surgery and Dentistry, University of Salerno, Italy

[2]IDC Hermitage-Capodimonte, Naples, Italy

[3]University of Naples "Federico II", Department of Advanced Biomedical Sciences, Naples, Italy

[4]Istituti Clinici Scientifici Maugeri IRCCS, Pavia, Italy

[5]Azienda Ospedaliera Universitaria OO. RR. San Giovanni di Dio e Ruggi d'Aragona, Salerno, Italy

[6]University of Naples "Federico II", Department of Chemical, Materials and Production Engineering, Naples, Italy

[7]University of Naples "Federico II", Department of Electrical Engineering and Information Technology, Naples, Italy

***Corresponding Author**:

Dr. Marianna Amboni

Department of Medicine, Surgery and Dentistry "Scuola Medica Salernitana", University of Salerno

Via Salvador Allende, 43, 84081 Baronissi (Sa), Italy

Phone: +39 089 968838

Email: marianna.amboni@gmail.com

Table S1. Univariate statistical analysis for comparing single and dual tasks in PD and PSP patients

PD			PSP		
**Features**	**Single task**<br>**Dual task**<br>**[Mean ± Dev. Std.]**	**p-value**	**Features**	**Single task**<br>**Dual task**<br>**[Mean ± Dev. Std.]**	**p-value**
Duration of the cycle^	1.10 ± 0.11<br>1.16 ± 0.14	**<0.0001*** **	Duration of the cycle^	1.29 ± 0.23<br>1.42 ± 0.28	**0.003***
Duration of the stance^	0.67 ± 0.08<br>0.72 ± 0.09	**<0.0001*** **	Duration of the stance^	0.82 ± 0.16<br>0.94 ± 0.22	**0.001****
Duration of the swing	0.37 ± 0.10<br>0.26 ± 0.08	**<0.0001*** **	Duration of the swing	0.27 ± 0.08<br>0.26 ± 0.05	0.856
Variability of swing's duration	0.07 ± 0.06<br>0.13 ± 0.04	**<0.0001*** **	Variability of swing's duration	0.17 ± 0.13<br>0.16 ± 0.04	0.573
Phase of stance^	60.27 ± 1.97<br>61.95 ± 2.20	**<0.0001*** **	Phase of stance	62.84 ± 2.79<br>65.64 ± 3.51	**0.001****
Phase of swing^	39.54 ± 1.70<br>38.54 ± 3.62	**0.011***	Phase of swing	37.01 ± 2.89<br>34.36 ± 3.51	**0.002***
Phase of single support^	39.45 ± 2.12<br>38.08 ± 2.54	**<0.0001*** **	Phase of single support	37.05 ± 2.95<br>34.36 ± 3.51	**0.003***
Phase of double support	10.82 ± 2.95<br>12.64 ± 3.10	**<0.0001*** **	Phase of double support	15.05 ± 6.58<br>17.47 ± 5.02	**0.048***
Mean velocity	1.04 ± 0.16<br>0.89 ± 0.19	**<0.0001*** **	Mean velocity	0.68 ± 0.26<br>0.52 ± 0.23	**0.001****
Mean velocity (%height)	62.07 ± 10.31<br>53.58 ± 10.95	**<0.0001*** **	Mean velocity (%height)^	41.69 ± 15.00<br>31.79 ± 13.53	**<0.0001*** **
Cadence^	109.61 ± 11.52	**<0.0001*** **	Cadence^	95.80 ± 17.27	**0.004****

	104.63 ± 12.30			87.99 ± 17.51	
Length of cycle^	1.13 ± 0.14<br>1.02 ± 0.17	**<0.0001*** **	Length of cycle	0.84 ± 0.21<br>0.69 ± 0.22	**<0.0001*** **
Length of cycle (%height)^	67.77 ± 8.82<br>61.47 ± 10.79	**<0.0001*** **	Length of cycle (%height)	51.70 ± 12.54<br>42.59 ± 12.95	**<0.0001*** **
Length of the step	0.46 ± 0.15<br>0.33 ± 0.10	**<0.0001*** **	Length of the step	0.25 ± 0.13<br>0.24 ± 0.17	**0.033***
Variability of the length of step	0.27 ± 0.42<br>0.24 ± 0.20	0.059	Variability of the length of step	0.31 ± 0.51<br>0.21 ± 0.30	0.709
Width of the step	0.10 ± 0.07<br>0.11 ± 0.10	0.675	Width of the step	0.11 ± 0.04<br>0.12 ± 0.06	**0.021***

*significance level at 0.05, **significance level at 0.01, ***significance level at 0.001. ^normally distributed according to the Kolmogorov Smirnov test; paired sample t-test applied. A Wilcoxon test was used to analyze non-normally distributed paired sample data.

Table S2. Univariate statistical analysis for comparing single and dual tasks in newly diagnosed PD and early PSP patients

**Newly diagnosed PD**			**Early PSP**		
**Features**	**Single task**<br>**Dual task**<br>**[Mean ± Dev. Std.]**	**p-value**	**Features**	**Single task**<br>**Dual task**<br>**[Mean ± Dev. Std.]**	**p-value**
Duration of the cycle	1.12 ± 0.13<br>1.17 ± 0.11	**0.003****	Duration of the cycle^	1.34 ± 0.24<br>1.45 ± 0.24	**0.033***
Duration of the stance	0.68 ± 0.10<br>0.73 ± 0.09	**0.001****	Duration of the stance^	0.85 ± 0.17<br>0.95 ± 0.19	**0.015***
Duration of the swing^	0.44 ± 0.04<br>0.44 ± 0.03	0.555	Duration of the swing^	0.49 ± 0.07<br>0.49 ± 0.07	0.926
Variability of swing's duration	0.03 ± 0.02<br>0.04 ± 0.02	0.737	Variability of swing's duration	0.12 ± 0.26<br>0.06 ± 0.02	0.347
Phase of stance^	60.48 ± 1.82<br>61.93 ± 1.76	**<0.0001*****	Phase of stance^	63.13 ± 2.38<br>65.62 ± 3.29	**0.006****
Phase of swing^	39.52 ± 1.82<br>38.07 ± 1.76	**<0.0001*****	Phase of swing^	36.78 ± 2.30<br>34.38 ± 3.29	**0.007****
Phase of single support^	39.49 ± 1.80<br>38.09 ± 1.76	**<0.0001*****	Phase of single support^	36.88 ± 2.38<br>34.35 ± 3.32	**0.006****
Phase of double support	11.33 ± 3.38<br>12.04 ± 1.75	**0.004****	Phase of double support^	13.05 ± 2.24<br>16.11 ± 3.82	**0.004****
Mean velocity	1.04 ± 0.18<br>0.91 ± 0.16	**<0.0001*****	Mean velocity	0.73 ± 0.29<br>0.58 ± 0.26	**0.004****
Mean velocity (%height)^	62.38 ± 11.66<br>55.24 ± 9.29	**<0.0001*****	Mean velocity (%height)	44.31 ± 16.89<br>35.34 ± 15.52	**0.004****
Cadence^	108.65 ± 11.45<br>103.52 ± 9.90	**<0.0001*****	Cadence^	92.47 ± 16.93<br>85.68 ± 15.38	**0.035***

Length of cycle^	1.14 ± 0.14<br>1.06 ± 0.12	**<0.0001*** **	Length of cycle^	0.92 ± 0.20<br>0.78 ± 0.22	**<0.0001*** **
Length of cycle (%height)^	68.54 ± 8.04<br>63.75 ± 7.09	**<0.0001*** **	Length of cycle (%height)^	56.17 ± 11.66<br>47.99 ± 13.38	**<0.0001*** **
Length of the step	0.53 ± 0.12<br>0.50 ± 0.10	**0.007** **	Length of the step^	0.43 ± 0.13<br>0.37 ± 0.14	**<0.0001*** **
Variability of the length of step	0.21 ± 0.42<br>0.20 ± 0.34	0.456	Variability of the length of step	0.25 ± 0.71<br>0.30 ± 0.77	**0.002** **
Width of the step	0.11 ± 0.10<br>0.10 ± 0.08	0.479	Width of the step^	0.08 ± 0.02<br>0.10 ± 0.03	**0.041***

*significance at 0.05, **significance at 0.01, ***significance at 0.001. ^normally distributed according to the Shapiro Wilk test; paired sample t-test applied. A Wilcoxon test was used to analyze non-normally distributed paired sample data.

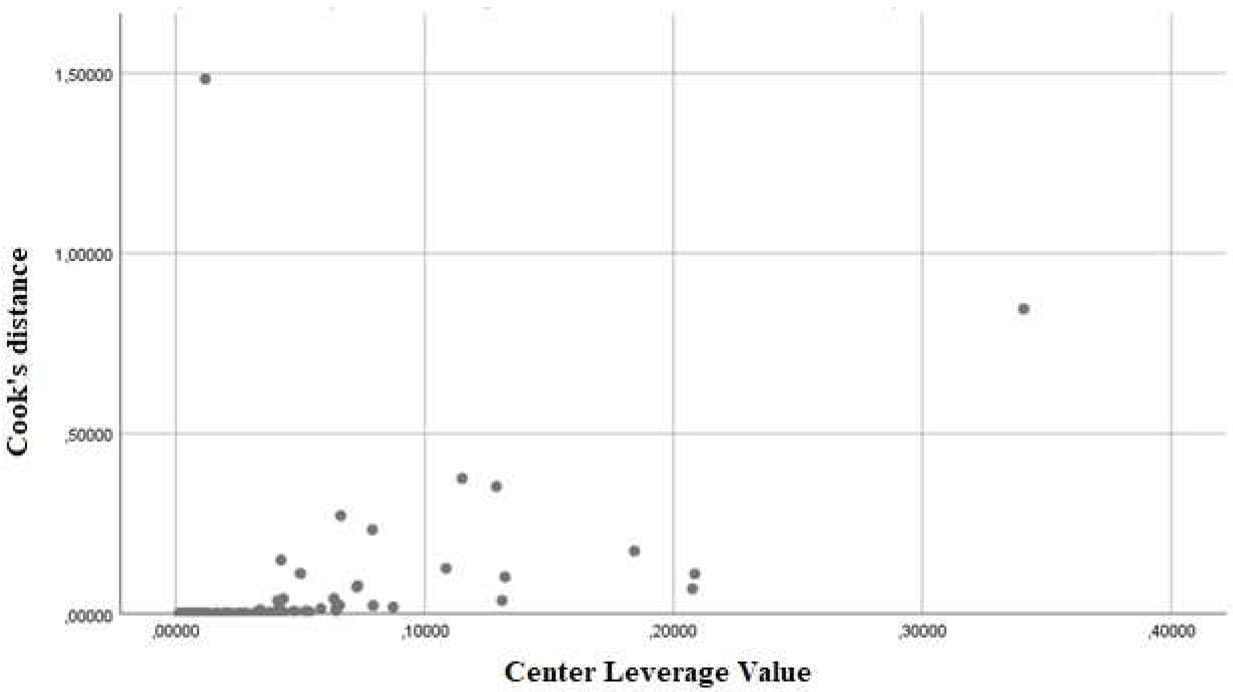

Figure S1. Cook's distance versus center leverage value for the GAIT model in PD vs PSP patients. Two outliers were removed.

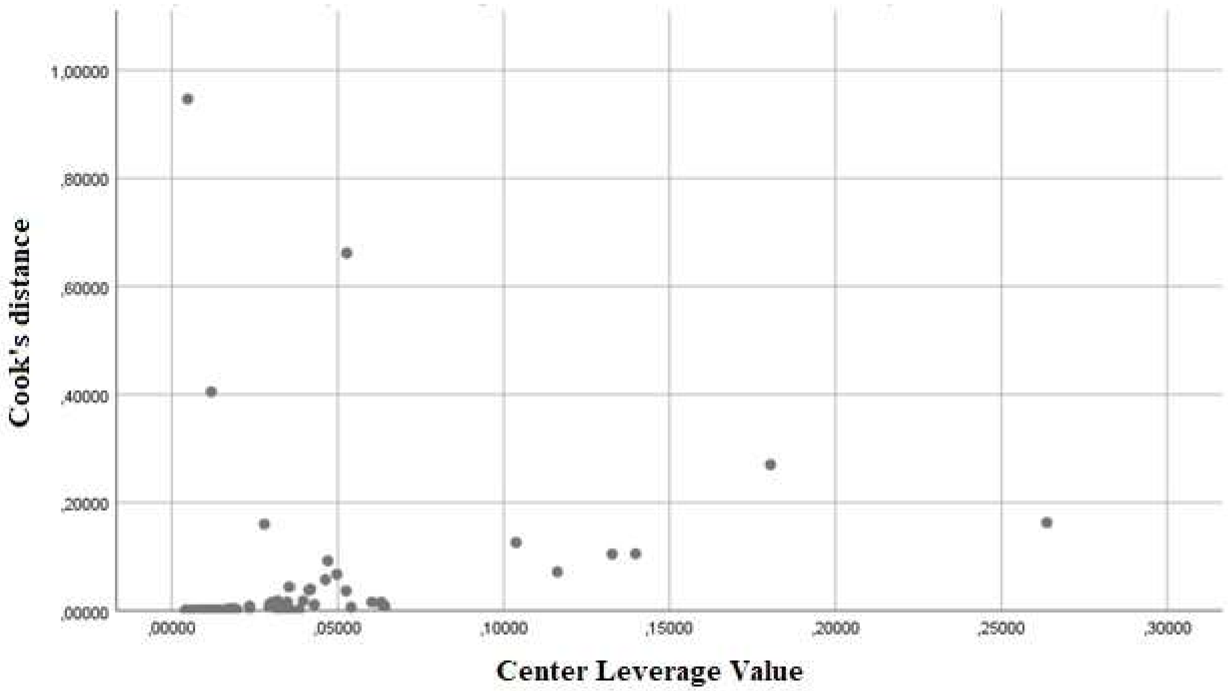

Figure S2. Cook's distance versus center leverage value for the COG model in PD vs PSP patients. Five outliers were removed.

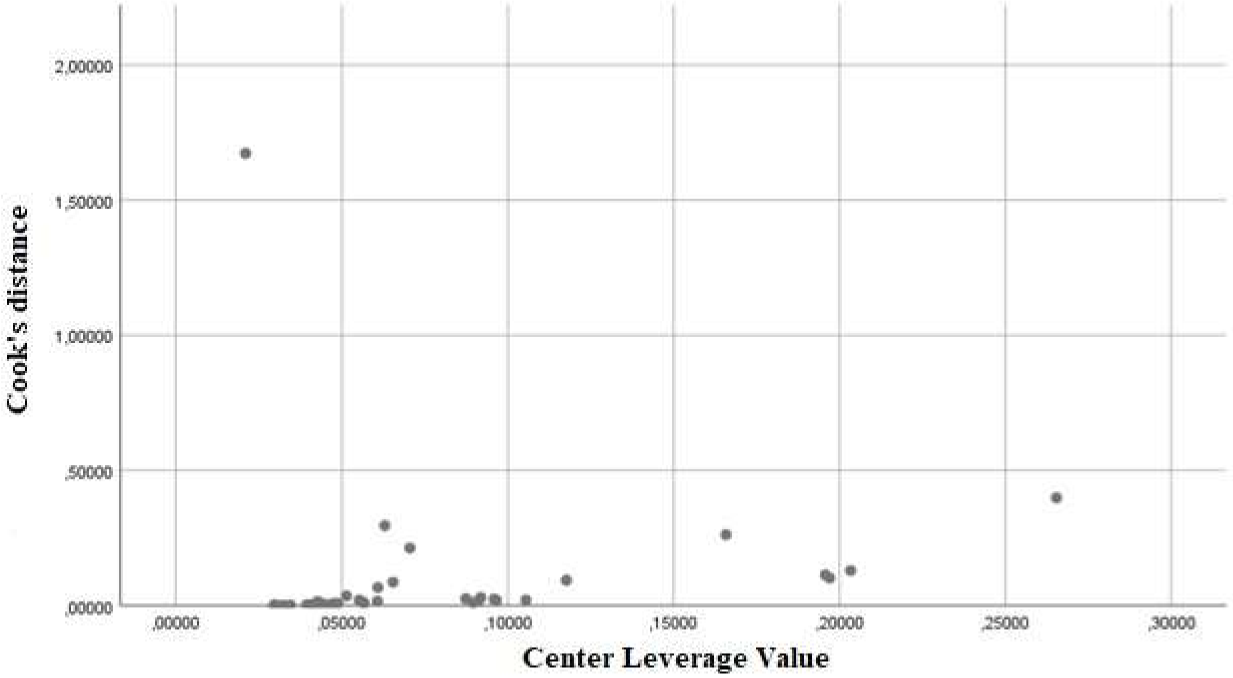

Figure S3. Cook's distance versus center leverage value for the GAIT model in newly diagnosed PD vs early PSP patients. Two outliers were removed.

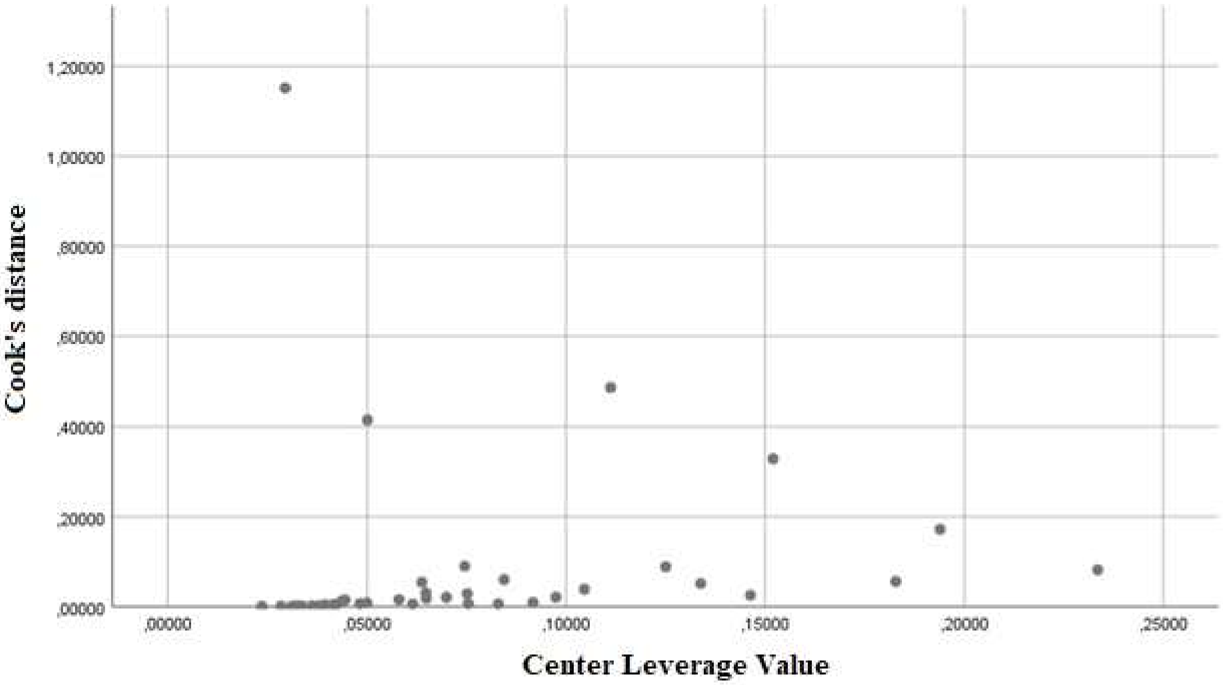

Figure S4. Cook's distance versus center leverage value for the COG model in newly diagnosed PD versus early PSP patients. Three outliers were removed.
